# Exposure to 100% Oxygen Abolishes the Impairment of Fracture Healing after Thoracic Trauma

**DOI:** 10.1371/journal.pone.0131194

**Published:** 2015-07-06

**Authors:** Julia Kemmler, Ronny Bindl, Oscar McCook, Florian Wagner, Michael Gröger, Katja Wagner, Angelika Scheuerle, Peter Radermacher, Anita Ignatius

**Affiliations:** 1 Institute of Orthopaedic Research and Biomechanics, Center of Musculoskeletal Research, University of Ulm, Ulm, Germany; 2 Institute of Pathophysiological Anaesthesiology and Process Engineering, Ulm University Medical Center, Ulm, Germany; 3 Department of Pathology, Ulm University Medical Center, Ulm, Germany; University of Zaragoza, SPAIN

## Abstract

In polytrauma patients a thoracic trauma is one of the most critical injuries and an important trigger of post-traumatic inflammation. About 50% of patients with thoracic trauma are additionally affected by bone fractures. The risk for fracture malunion is considerably increased in such patients, the pathomechanisms being poorly understood. Thoracic trauma causes regional alveolar hypoxia and, subsequently, hypoxemia, which in turn triggers local and systemic inflammation. Therefore, we aimed to unravel the role of oxygen in impaired bone regeneration after thoracic trauma. We hypothesized that short-term breathing of 100% oxygen in the early post-traumatic phase ameliorates inflammation and improves bone regeneration. Mice underwent a femur osteotomy alone or combined with blunt chest trauma 100% oxygen was administered immediately after trauma for two separate 3 hour intervals. Arterial blood gas tensions, microcirculatory perfusion and oxygenation were assessed at 3, 9 and 24 hours after injury. Inflammatory cytokines and markers of oxidative/nitrosative stress were measured in plasma, lung and fracture hematoma. Bone healing was assessed on day 7, 14 and 21. Thoracic trauma induced pulmonary and systemic inflammation and impaired bone healing. Short-term exposure to 100% oxygen in the acute post-traumatic phase significantly attenuated systemic and local inflammatory responses and improved fracture healing without provoking toxic side effects, suggesting that hyperoxia could induce anti-inflammatory and pro-regenerative effects after severe injury. These results suggest that breathing of 100% oxygen in the acute post-traumatic phase might reduce the risk of poorly healing fractures in severely injured patients.

## Introduction

More than 30% of polytrauma patients experience fractures of the extremities [1]. In these patients bone healing is considerably disturbed, and the risk for malunion increases up to 50% depending on trauma severity [2, 3]. Severe trauma generates a systemic inflammatory response, due to cell and matrix debris and cytosolic molecules (e.g. adenosine triphosphate) that are released from injured tissues and act as endogenous alarm signals [4]. The post-traumatic systemic inflammation is characterized by activation of immune cells, stimulation of protein cascades including the complement and coagulation systems, and a burst of pro- and anti-inflammatory mediators [5]. This inflammatory reaction can impair cell function even in remote tissues not directly affected by the initial trauma, and may consequently lead to multi-organ dysfunction [6]. It is, therefore, evident that the over-activated immune response after severe trauma also accounts for impaired bone regeneration, although the cellular and molecular mechanisms are far from being well understood.

Fracture healing is a complex process with closely linked phases of inflammation, repair and remodeling. Here, we focused on the acute inflammatory phase, which is characterized by the invasion of immune cells and the production of pro- and anti-inflammatory mediators in the fracture hematoma [7, 8]. A balanced local inflammation is important for subsequent bone regeneration, and even small perturbations of the early inflammatory phase, e.g. due to hyper-inflammation after polytrauma, may impair the healing process [7, 9, 10].

Despite the high clinical relevance, so far few studies addressed the problem of compromised bone healing in polytrauma. Our group established an experimental model of severe trauma with concomitant femur fracture in rodents [11]. For severe trauma we chose a blunt thoracic trauma, due to its high clinical relevance – 50% of polytrauma patients suffer from thoracic trauma [1] – and, because it induces a strong systemic inflammation [12]. Confirming results of clinical studies [2, 3], the additional thoracic trauma markedly impaired fracture healing in this model [11, 13, 14]. It was previously shown in this combined injury model that blocking the complement system, a critical trigger of post-traumatic inflammation, attenuated the deleterious effects on bone regeneration by suppressing the systemic immune response [14].

Blunt chest trauma may lead to acute lung injury, resulting in regional alveolar hypoxia and subsequent hypoxemia and tissue hypoxia, which in turn triggers systemic and local hyper-inflammation [15, 16]. Even in the absence of any mechanical injury, alveolar hypoxia induces invasion of immune cells into the lung and increases the expression of inflammatory transcription factors (e.g. *nuclear factor-κB* (NF-κB)) and of pro- and anti-inflammatory cytokines [17]. Moreover, neutrophil function is disturbed by acute hypoxia, demonstrating that transient hypoxemic insults trigger hyper-inflammation [18]. Hyperoxia, i.e. increasing the inspired oxygen (O_2_) concentration, can counteract alveolar hypoxia and in turn hypoxemia and tissue hypoxia [19], ultimately modulating hyper-inflammation: in fact, short-term ventilation with 100% O_2_ reduced inflammation and apoptosis in various shock models [20, 21]. However, pure O_2_ breathing may further aggravate the inflammatory response as a result of increased oxidative and nitrosative stress [22].

The present study aimed to unravel the role of O_2_ tension in the pathogenesis of impaired fracture healing after thoracic trauma. Using a mouse model of combined femur fracture and thoracic trauma we addressed the following questions: 1) Is impaired bone healing after thoracic trauma triggered by hypoxemia, and 2) Does intermitted exposure to 100% O_2_ in the acute post-traumatic phase ameliorate the inflammatory response and thereby improve fracture healing?

## Materials and Methods

### Study design

All animal experiments were approved by the local ethical committee (Regierungspräsidium Tübingen, Germany) and were performed in accordance with the international regulations for the care and use of laboratory animals. Male, 12-week aged C57BL/6 mice were purchased from Charles River (Sulzfeld, Germany). Mice received a standard mouse feed (ssniff R/M-H, V1535-300, Ssniff GmbH, Soest, Germany) and water *ad libitum*.

All mice were randomly assigned to three groups: isolated femur fracture (F); fracture combined with blunt thoracic trauma (F+TXT); fracture, thoracic trauma and O_2_ treatment (F+TXT+O_2_).

Arterial blood gas tensions, acid-base status, parameters of microcirculatory perfusion and oxygenation were determined at 3, 9 and 24 hours after surgery. Pro- and anti-inflammatory cytokines/chemokines and markers of oxidative and nitrosative stress were measured in plasma, lung and fracture callus. Bone healing was assessed by (immuno-) histomorphometry, micro-computed tomography (μCT), and biomechanical analyses on day 7, 14 and 21. Animals were sacrificed after 3, 9 and 24 hours and 3, 7, 14 and 21 days under general anaesthesia through blood withdrawal via the vena cava inferior. During blood withdrawal, the fraction of inspired O_2_ (FiO_2_) was 0.35.

### Femur osteotomy

Mice were anesthetized with 2% isoflurane (Forene, Abbott, Wiesbaden, Germany) in 100% O_2_. An osteotomy gap (0.4 mm) was created at the mid-shaft of the right femur and stabilized using an external fixator (axial stiffness 3 N/mm, RISystem, Davos, Switzerland) as described previously [23]. Briefly, the external fixator consists of a stiff polymeric block and four mini-Schanz screws with a diameter of 0.45 mm.

Before surgery, all animals received a single dose of antibiotic (clindamycin-2-dihydrogenphosphate, 45 mg/kg, Clindamycin, Ratiopharm, Ulm, Germany). For analgesia, tramadol hydrochloride (Tramal, Gruenenthal GmbH, Aachen, Germany) was provided via the drinking water, starting 1 day prior to surgery until 3 days post-surgery.

### Thoracic trauma

The thoracic trauma was applied immediately after fracture while the mice were still under general anaesthesia. In brief, a single blast wave was applied on the middle of the thorax using a blast wave generator, which was centered 2 cm above the thorax. This model allows a standardized bilateral, isolated lung contusion [24].

### O_2_ treatment

Mice subjected to intermittent O_2_ treatment were exposed to 100% O_2_ directly after surgery for two 3 hours intervals, with a period of 3 hours of normal ambient air in between. For 100% O_2_ exposure mice were placed into chambers with constant O_2_ influx. O_2_ concentration was continually recorded using an oxymeter. Control mice constantly breathed air.

### Blood gas analysis and hemodynamic parameters

Arterial blood (50 μl) was collected for measurements of the partial pressure of oxygen (pO_2_), and carbon dioxide (pCO_2_) and pH (ABL 700, Diamond Diagnostics, Holliston, MA, USA) 3, 9 and 24 hours post-surgery. Microvascular blood flow (recorded in arbitrary units) and the haemoglobin O_2_ saturation (μHb-O_2_) were determined at the fracture site by laser Doppler flowmetry and simultaneous remission spectroscopy (O_2_C, LEA Medizintechnik, Gießen, Germany) as described previously [25]. Briefly, via the power spectra of backscattered laser light that incorporates the scattering of Doppler shifts of the erythrocyte velocities, the microvascular blood flow was acquired. An incision was made to gain access to the fracture gap and the probe was directly placed near the gap. Three separate measurements were performed and the mean value was calculated.

### Comet assay

20 μl blood was used to perform the alkaline version of the comet assay, to detect deoxyribonucleic acid (DNA) damage [26]. In brief, cells were denaturized with alkali (pH 13) for 30 minutes, followed by electrophoresis for 25 minutes at 25 V and 300 mA. After staining with ethidium bromide, the tail moment of 50 randomly chosen nuclei were determined by image analysis (Comet Assay IV, Perceptive Instruments, Haverhill, UK)

### Cytokine analysis in blood plasma and lung homogenates

After final blood withdrawal 3, 9 and 24 hours post-surgery, blood and lung tissue was harvested for cytokine analysis. Blood was collected in microvettes (Sarstedt AG & Co., Nümbrecht, Germany), centrifuged at 4000 g for 10 minutes, followed by another centrifugation step at 10.000 g for 1 minutes before plasma was collected and stored at -80°C. The lung was frozen in liquid nitrogen and stored at -80°C. Then the tissue was homogenized and the cells were lysed on ice (30 minutes) and centrifuged before the supernatant was collected. The protein concentration was set at 100 μg. Both in plasma and lung homogenates concentrations of tumor necrosis factor (TNF)-α, interleukin (IL)-1β, IL-6, IL-10, monocyte chemotactic protein (MCP)-1 and keratinocyte chemoattractant (KC) were measured by a mouse multiplex cytokine kit according to the manufacturer’s protocol (Bio-Plex Pro Cytokine Assay, Bio-Rad, Hercules, CA). The analysis was performed in duplicates. Data were automatically analysed using the standard curve of cytokine standards (Bio-Plex Manager Software 4.1). Values below the detection limit of the assay were set to zero.

### Immunoblots

To determine heme oxygenase (HO-1) in protein extracts (20–40 μg) of lung homogenates (protein extraction described under “cytokine analysis”), samples were separated by sodium dodecyl sulfate polyacrylamide gel electrophoresis and transferred to a nitrocellulose membrane. After blocking with 5% dry milk in PBS containing 0.1% Tween-20, the primary antibody (monoclonal anti-HO-1, Abcam, Cambridge, United Kingdom, dilution 1:2000) was applied. For detection, a horseradish peroxidase (HRP)-conjugated secondary antibody (goat anti-rabbit, Cell Signaling Technology, Schwerte, Germany or Santa Cruz, Heidelberg, Germany; dilution: 1:15000) was used. Membranes were exposed to chemiluminescence (SuperSignal West Femto Maximum Sensitivity Substrate, Thermo Scientific, Dreieich, Germany), before films were scanned. Branch intensities of immunoreactivity were assessed by NIH ImageJ software (http://rsb.info.nih.gov/nih-image). Membranes were incubated with an anti β-actin antibody as reference. To allow direct comparisons between individual gels after immunoblotting, samples of control animals, which had not undergone surgery or trauma, were loaded simultaneously on each gel. Intensities of each band were related to the control samples. Data were expressed as fold increase over control.

### Histomorphometry of lungs and fracture calli

On day 1, 3 and 21, left lungs were fixed in 10% formalin and embedded in paraffin. Hematoxylin-eosin-stained sections were used to analyse overall lung injury, dystelectasis, alveolar wall thickening, emphysema, blood clotting and inflammation by light microscopy (Leica DMI6000 B, Leica, Heerbrugg, Switzerland). The parameters were scored on a scale of 0–4: 0 for normal lungs, 1 for minor damage (≤ 25%), 2 for moderate damage (25–50%), 3 for considerable damage (50–75%) and 4 for strong damage ≥ 75%. The “total lung pathology score” represents the sum score of all parameters [27].

Femurs collected 14 days post-surgery were demineralized, embedded in paraffin and subjected to Safranin-O staining. Bone specimens from 21 days were used for undecalcified histology, embedded in methyl methacrylate and Giemsa stained. Longitudinal sections were evaluated by light microscopy and contents of bone, cartilage and fibrous tissue were determined using an image analysing software (MMAF Version 1.4.0 MetaMorph, Leica). The region of interest was defined as the periosteal callus between the inner pins of the fixator, including the fracture gap.

### Immunohistochemistry of lungs

Paraffin embedded lung sections were subjected to immunohistological staining for neutrophils (Ultra-LEAF Purified rat anti-mouse Ly-6G, BioLegend, San Diego, CA, USA), Caspase-3 (Cleaved Caspase-3 (Asp175), Cell Signalling Technology, Schwerte, Germany) and nitrotyrosine (anti-nitrotyrosine: AB5411, Merck Millipore, Schwalbach, Germany). Lungs were deparaffinised in xylene, rehydrated in ethanol and then boiled twice in sodium citrate buffer for antigen retrieval. Sections were blocked with 10% goat serum for 30 minutes and incubated with the primary antibodies (Ly6-G 1:200; Caspase-3 1:100; anti-nitrotyrosin 1:200) for 1 hours at room temperature. Secondary antibodies (goat anti-rabbit IgG (H+L) Alk. Phos., 1:40, Jackson Immuno Research Laboratories Inc., Baltimore, PA, USA; or goat anti-rat IgG Alk. Phos., 1:100, AbD Serotec, Oxford, United Kingdom) were added for 30 minutes. Antibodies were detected using alkaline phosphate red chromogen (Dako REAL Detection System; Dako Corp, Hamburg, Germany), followed by counterstaining with haematoxylin. For evaluation of the neutrophil number, positive cells were counted in the whole tissue sample. To analyse nitrotyrosine and caspase-3 staining, four representative fields per section (800 μm^2^) were quantified for intensity and percent immunoreactive regions (Axio Vision software, Zeiss, Jena, Germany). Results represent the mean densitometric sum red.

### Immunohistochemistry of fracture calli

Immunohistological stainings were performed on days 1, 3, 7 and 14 post-surgery. Femurs were stained for macrophages (F4/80, Bio-Rad AbD Serotec GmbH, Puchheim, Germany), IL-6 (Bioss Inc, Woburn, MA, USA), IL-10 (Bioss Inc) and platelet endothelial cell adhesion molecule 1 (PECAM-1) (DIA-310, Dianova GmbH, Hamburg, Germany). Additional neutrophil and nitrotyrosine stainings were performed as described for lung tissue. Bone specimens were deparaffinised and rehydrated, followed by blocking in 5% goat serum for 1 hour at room temperature. Primary antibodies (macrophages 1:500; IL-6 1:250; IL-10 1:250; PECAM-1 1:10) were incubated overnight at 4°C. The secondary antibody, either a biotinylated goat-anti-rabbit antibody (Invitrogen, Life Technologies Corporation, Darmstadt, Germany; for IL-6, IL-10) or a goat-anti-rat antibody (Invitrogen; for macrophages and PECAM-1) was added for 30 minutes at room temperature. For signal detection in IL-6, IL-10 and PECAM-1 stainings, Vectastain Elite ABC kit and Vector NovaRED substrate (both Vector laboratories Inc., Burlingame, CA, USA) were applied according to the manufacturer’s protocol. Signal amplification in macrophage stainings was achieved using HRP-linked streptavidin (15 minutes) and subsequent AEC Single Solution (both Zytomed, Berlin, Germany). All sections were counterstained using haematoxylin. The region of interest was defined as the periosteal callus between the inner pins of the fixator, excluding the fracture gap. Macrophage stainings were examined in the marrow cavity at the fracture site.

### Biomechanical testing

To evaluate the mechanical competence of the healed bone, the mechanical properties of fractured femurs explanted on day 21 were investigated using a non-destructive three-point bending test on a material testing machine (1454, Zwick GmbH, Ulm, Germany). The method was previously described in detail [23]. Briefly, after careful removal of the fixator, femurs were fixed in aluminium cylinders, which were then fixed in the testing machine, with the condyles unfixed on a distal bending support. An axial load was applied to the top of the callus at the femoral midshaft, with a maximum force of 4 N. The flexural rigidity (EI) was calculated using the formula EI = k(a^2^b^2^/3L) in N/mm^2^ with a = the distance between load application and distal bending support, b = the distance between load application and proximal bending support, k = slope of the linear region of the force-deflection curve and L = length between both bending supports(a, b) [28].

### Micro-computed tomography (μCT)

After biomechanical testing, fracture calli were scanned by a μCT device (Skyscan 1172, Skyscan, Kontich, Belgium) to evaluate bone formation and structural properties of the fracture calli at 8 μm resolution using a voltage of 50 kV and 200 μA. Calibration and global thresholding (641.9 mg hydroxyapatite/cm^3^ for callus and cortical bone; 394.8 mg hydroxyapatite/cm^3^ for trabecular bone) were performed as described before [29]. The ratio of bone volume to tissue volume (BV/TV) and the maximum moment of inertia (I_x_) of the callus in orientation to the bending axis were determined. Using EI and I_x_, the apparent Young’s modulus (E_app_) was calculated according E_app_ = (EI/I_x_).

### Statistical analysis

In all figures, results are depicted as box and whiskers plots, showing medians, upper and lower quartiles, maximum and minimum. Outliers were marked as circles. Data in tables show median and quartiles. Statistical evaluation was performed using IBM SPSS Statistics software 19.0 (SPSS Inc., Chicago, USA). Groups were tested for normal distribution using Shapiro-Wilk test, and then compared by either Kruskall-Wallis and Dunn’s *post-hoc* test, or by one-way ANOVA and Fishers LSD *post-hoc* test. The level of significance was set at p≤ 0.05. We used at least 5 mice per group and time point. At some observation time points, additional animals were included, because we could not perform all analyses on the same tissue specimens due to technical reasons (e.g. histology vs. tissue homogenates). Because we evaluated all available tissues from those additional animals, the number of samples per evaluated parameter was sometimes higher. The sample numbers were indicated in tables and figures.

## Results

### O_2_ treatment induces hyperoxia

Table 1 summarizes the data of the arterial blood gas analysis, the acid-base-status, oxygenation as well as microcirculatory perfusion at the fracture site. pO_2_ in arterial blood was only slightly decreased at 3 hours after thoracic trauma in comparison to mice with isolated fracture, whereas the pCO_2_ and pH were hardly affected, indicating only moderate overall lung injury. O_2_ treatment significantly increased pO_2_ when compared to normoxia groups with isolated fracture (+48%, p = 0.014) or combined trauma (+74%, p = 0.001). Later observation time points did not show any significant intergroup differences. While the thoracic trauma had no further effect on μHb-O_2_ values in mice with fracture alone, μHb-O_2_ was significantly increased after O_2_ treatment 3 hours (+34%, p = 0.013) and 9 hours (+25%, p = 0.019) post-injury. Microcircular blood flow measured at the fracture gap did not differ between the groups.

**Table 1:** Blood gas analysis of arterial blood, acid-base status, hemoglobin oxygen saturation and microvascular blood flow 3, 9 and 24 hours post-injury.

		F	F+TXT	F+TXT+O_2_
**pO**_**2**_ **(mmHg)**	3 h	142 (67; 150) (n = 5)	87 (75; 94) (n = 5)	162 (143; 211)* ## (n = 8)
	9 h	165 (107; 180) (n = 5)	121 (87; 139) (n = 5)	136 (75; 196) (n = 7)
	24 h	173 (93; 192) (n = 10)	111 (66; 202) (n = 13)	187 (119; 203) (n = 17)
**pCO**_**2**_ **(mmHg)**	3 h	24 (22; 31) (n = 5)	30 (28; 47) (n = 5)	26 (19; 35) (n = 8)
	9 h	32 (25; 39) (n = 5)	30 (27; 35) (n = 5)	29 (25; 36) (n = 7)
	24 h	27 (24; 34) (n = 10)	33 (28; 35) (n = 13)	30 (27; 34) (n = 17)
**pH**	3 h	7.30 (7.24; 7.38) (n = 5)	7.23 (7.19; 7.31) (n = 5)	7.28 (7.23; 7.38) (n = 8)
	9 h	7.25 (7.25; 7.31) (n = 5)	7.35 (7.32; 7.36) (n = 5)	7.24 (7.21; 7.33) (n = 7)
	24 h	7.34 (7.30; 7.40) (n = 10)	7.28 (7.27; 7.36) (n = 13)	7.30 (7.25; 7.36) (n = 17)
**μHb-O**_**2**_ **(%)**	3 h	54 (41; 67) (n = 5)	61 (57; 72) (n = 5)	77 (62; 83)* (n = 9)
	9 h	57 (49; 62) (n = 5)	63 (51; 80) (n = 5)	69 (69; 73)* (n = 7)
	24 h	73 (63; 77) (n = 14)	72 (66; 76) (n = 16)	75 (71; 78) (n = 17)
**Blood flow (AU)**	3 h	34 (27; 43) (n = 9)	33 (24; 59) (n = 8)	25 (18; 65) (n = 9)
	9 h	38 (18; 78) (n = 8)	29 (24; 50) (n = 8)	34 (27; 43) (n = 9)
	24 h	39 (27; 67) (n = 14)	43 (30; 61) (n = 16)	39 (28; 75) (n = 17)

Data represent medians and quartiles. Specimen numbers for each group are depicted.

*p < 0.05 vs F

##p < 0.01 vs. F+TXT

FiO_2_ was 0.35 during blood withdrawal.

### O_2_ treatment attenuates trauma induced systemic inflammation without provoking toxic side effects

Table 2 shows the concentrations of pro- and anti-inflammatory cytokines and chemokines in blood plasma and the results of the comet assay. Plasma cytokine levels obtained from mice with isolated fracture were not increased compared to sham mice of another study [30], indicating that the isolated fracture did not induce severe systemic inflammation. In comparison to fracture alone, the concomitant thoracic trauma significantly increased plasma concentrations of IL-6 (+196%, p < 0.001), IL-10 (+78%, p = 0.017) and MCP-1 (+65%, p = 0.008), 3 hours post-injury. Exposure to 100% O_2_ diminished IL-6, IL-10 and MCP-1 concentrations to normal levels.

**Table 2:** Cytokine/chemokine concentrations in blood plasma and tail moments.

Cytokine (pg/ml)		F	F+TXT	F+TXT+O_2_
IL-1β	3 h	21 (17; 27) (n = 8)	13 (9; 26) (n = 6)	23 (12; 40) (n = 8)
	9 h	21 (14; 29) (n = 10)	13 (4; 21) (n = 8)	23 (16; 25) (n = 9)
	24 h	18 (8; 26) (n = 8)	23 (5; 26) (n = 9)	17 (11; 24) (n = 9)
IL-6	3 h	49 (41; 54) (n = 8)	152 (64; 190)** (n = 6)	55 (46; 96)## (n = 9)
	9 h	45 (40; 54) (n = 10)	43 (30; 59) (n = 8)	38 (37; 57) (n = 9)
	24 h	9 (7; 11) (n = 8)	10 (8; 11) (n = 9)	8 (5; 18) (n = 9)
IL-10	3 h	27 (15; 34) (n = 8)	41 (32; 71)*b(n = 6)	22 (15; 29)## (n = 9)
	9 h	27 (23; 35) (n = 10)	20 (11; 31) (n = 8)	22 (16; 39) (n = 9)
	24 h	13 (9; 17) (n = 8)	11 (10; 20) (n = 9)	12 (9; 18) (n = 10)
TNF-α	3 h	37 (17; 50) (n = 8)	25 (23; 44) (n = 6)	36 (21; 61) (n = 9)
	9 h	43 (34; 52) (n = 10)	26 (12; 42)* (n = 8)	31 (25; 48) (n = 9)
	24 h	25 (9; 38) (n = 8)	32 (9; 48) (n = 9)	19 (9; 32) (n = 10)
KC	3 h	106 (72; 172) (n = 8)	51 (39; 160) (n = 6)	102 (36; 176) (n = 9)
	9 h	110 (69; 131) (n = 10)	49 (37; 74) (n = 8)	90 (52; 137) (n = 9)
	24 h	14 (10; 19) (n = 8)	20 (7; 26) (n = 9)	18 (8; 33) (n = 10)
MCP-1	3 h	94 (81; 104) (n = 8)	139 (102; 219)** (n = 6)	105 (90; 120)# (n = 8)
	9 h	76 (68; 81) (n = 10)	70 (59; 82) (n = 7)	85 (81; 108)** ## (n = 9)
	24 h	56 (49; 65) (n = 8)	71 (64; 85)* (n = 8)	73 (57; 92)* (n = 8)
Tail Moment	3 h	0.34 (0.28; 0.39) (n = 9)	0.36 (0.32; 0.40) (n = 8)	0.32 (0.29; 0.41) (n = 9)
	9 h	0.33 (0.30; 0.40) (n = 9)	0.39 (0.30; 0.47) (n = 9)	0.37 (0.33; 0.44) (n = 9)
	24 h	0.40 (0.33; 0.43) (n = 14)	0.42 (0.34; 0.52) (n = 16)	0.38 (0.33; 0.52) (n = 17)

Data represent medians and quartiles. Specimen numbers for each group are depicted.

*p < 0.05 and

**p < 0.01 vs F

#p < 0.05 and

##p < 0.01 vs. F+TXT.

**Fig 1:**
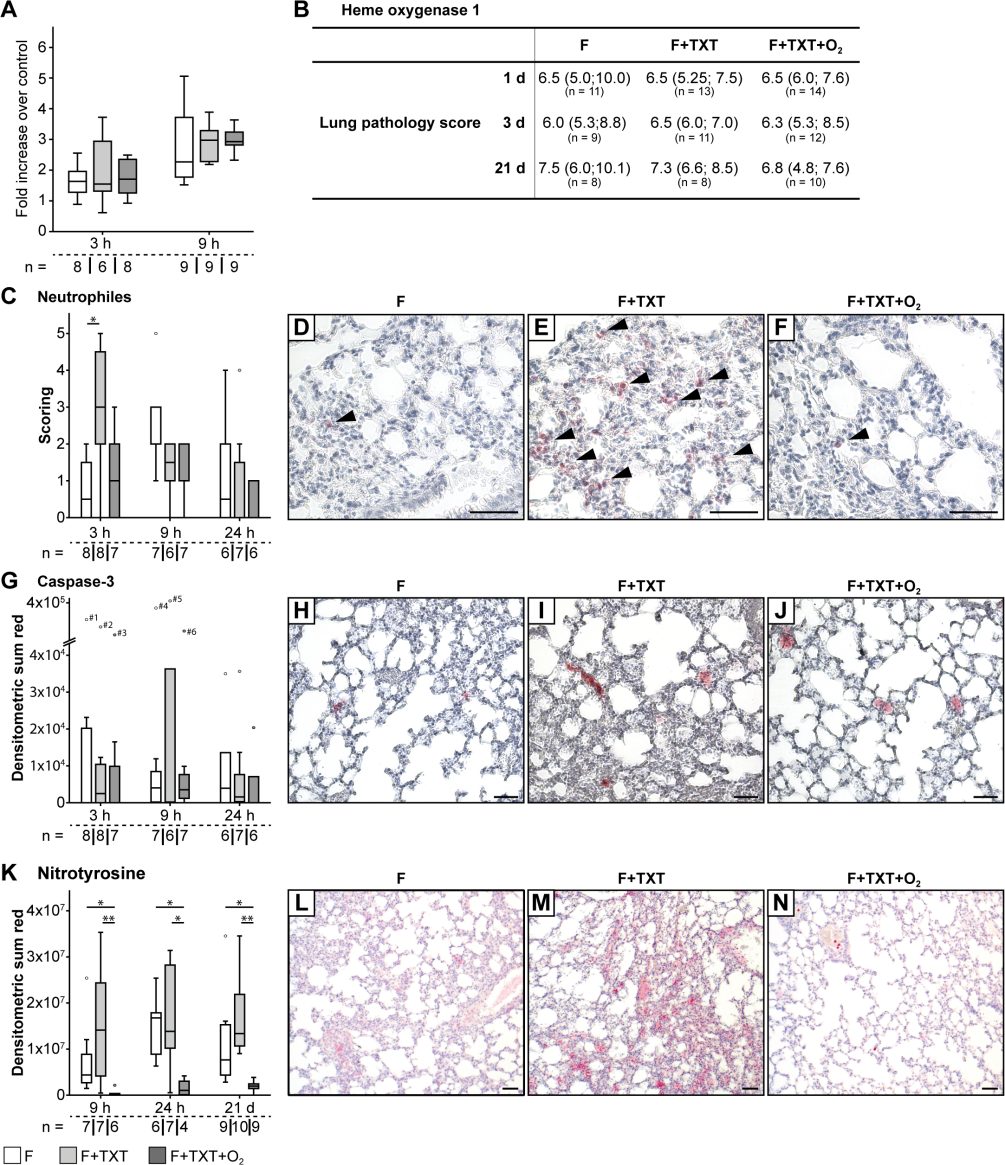
Microscopic and molecular analyses of lungs. (A) Lung tissue expression of HO-1 3 and 9 hours post-injury. (B) Quantitative analysis of lung histology after 1, 3 and 21 d. (C) Histological scoring of neutrophil stainings in the lungs and (D-F) representative slides 3 hours after injury showing increased numbers of neutrophils after TXT; arrows highlight positively stained neutrophils. (G) Analysis of caspase-3 staining and (H-J) representative slides of each group, 3 hours after injury. Values of outliers: #1 = 7.7x10^4^; #2 = 6.69x10^4^; #3 = 5.7x10^4^; #4 = 2.96x10^5^; #5 = 4.07x10^5^; #6 = 6.16x10^4^. (K-N) Positive nitrotyrosine staining was detected up to 21 days after surgery. O_2_ treatment significantly decreased the level of nitrotyrosine. Scale bars: 50 μm; box-plots represent medians and quartiles, whiskers represent the minimum and maximum values. Data represent medians and quartiles. Specimen numbers for each group are depicted. *p<0.05, **p < 0.001.

To exclude possible toxic side effects of the O_2_ treatment, we tested whole blood samples for DNA-strand breaks using the alkaline comet assay. Tail moments did not show any significant intergroup or time-dependent differences. HO-1 expression did not differ either following thoracic trauma or O_2_ exposure 3 and 9 hours after surgery, indicating the absence of additional oxidative stress (Fig 1A).

### O_2_ treatment reduces trauma induced lung inflammation

Fig 1B demonstrates that neither thoracic trauma nor O_2_ exposure significantly influenced the lung pathology score. However, the combined trauma under normoxic conditions significantly increased the invasion of neutrophils in the lung, 3 hours after injury, and O_2_ treatment completely abolished this effect (Fig 1C–1F). The combined trauma also significantly increased tissue concentrations of IL-1β (+30%, p = 0.008), IL-6 (+52%, p = 0.008) and IL-10 (+78%, p = 0.017) 3 hours after injury. Again, this effect was nearly abolished by O_2_ exposure (Table 3). While lung tissue caspase-3 expression did not show any significant intergroup differences throughout the experiment (Fig 1G–1J), hyperoxia decreased the otherwise marked nitrotyrosine formation until day 21, demonstrating the additional absence of nitrosative stress (Fig 1K–1N).

**Table 3:** Cytokine/chemokine concentrations in lung homogenates.

Cytokine (pg/ml)		F	F+TXT	F+TXT+O_2_
IL-1β	3 h	103 (92; 108) (n = 8)	143 (105; 151)** (n = 6)	114 (108; 126) (n = 8)
	9 h	117 (108; 123) (n = 10)	113 (101; 133) (n = 8)	109 (97; 119) (n = 9)
	24 h	127 (116; 151) (n = 8)	114 (103; 120) (n = 9)	112 (104; 118) (n = 8)
IL-6	3 h	3.0 (2.8; 3.4) (n = 8)	4.5 (3.2; 5.8)** (n = 6)	3.5 (2.9; 3.6)# (n = 8)
	9 h	3.2 (2.8; 3.7) (n = 10)	3.4 (3.1; 4.3) (n = 8)	3.4 (3.0; 3.8) (n = 9)
	24 h	4.9 (4.2; 5.8) (n = 8)	4.4 (4.1; 5.4) (n = 9)	4.5 (3.9; 4.7) (n = 7)
IL-10	3 h	27 (24; 30) (n = 8)	34 (26; 57)* (n = 6)	28 (25; 32)# (n = 8)
	9 h	32 (30; 41) (n = 10)	30 (27; 44) (n = 8)	31 (24; 38) (n = 9)
	24 h	30 (24; 41) (n = 8)	29 (24; 31) (n = 9)	23 (22; 28) (n = 8)
TNF-α	3 h	59 (56; 63) (n = 8)	58 (55; 90) (n = 6)	66 (56; 69) (n = 8)
	9 h	68 (61; 75) (n = 10)	62 (56; 67) (n = 8)	62 (56; 69) (n = 9)
	24 h	125 (114; 146) (n = 8)	111 (99; 121) (n = 9)	102 (98; 114)** (n = 8)
KC	3 h	151 (124; 178) (n = 8)	99 (87; 232) (n = 6)	159 (93; 182) (n = 8)
	9 h	106 (86; 142) (n = 10)	123 (82; 146) (n = 8)	105 (77; 133) (n = 9)
	24 h	27 (20; 56) (n = 8)	21 (15; 25) (n = 9)	25 (17; 43) (n = 8)
MCP-1	3 h	61 (57; 67) (n = 8)	62 (53; 68) (n = 6)	71 (55; 75) (n = 8)
	9 h	68 (67; 74) (n = 10)	65 (57; 71) (n = 8)	64 (61; 69) (n = 9)
	24 h	67 (63; 75) (n = 8)	66 (58; 71) (n = 9)	61 (57; 65)* (n = 8)

Data represent medians and quartiles. Specimen numbers for each group are depicted.

*p < 0.05 and

**p < 0.01 vs F

#p < 0.05 vs. F+TXT.

### O_2_ treatment attenuates impaired fracture healing induced by thoracic trauma

**Fig 2:**
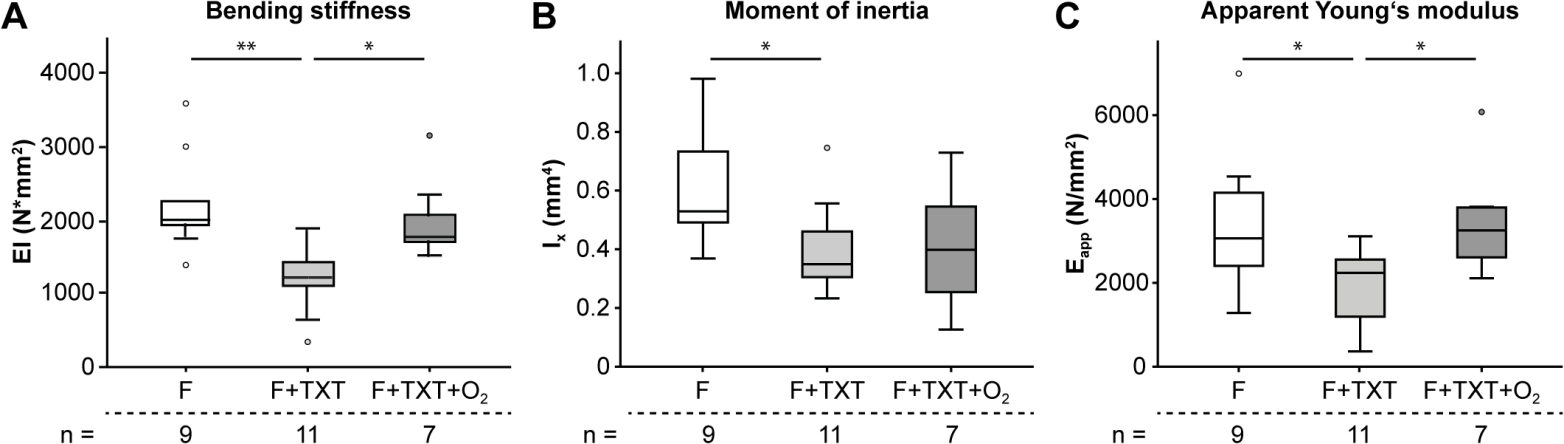
Biomechanical and μCT analysis of the fracture callus 21 days post-injury. (A-C) The binding stiffness, moment of inertia and the apparent Young’s modulus of the fracture callus were decreased following TXT, O_2_ treatment abolished these effects. Data represent medians and quartiles. Specimen numbers for each group are depicted. *p<0.05, **p < 0.001.

Fracture healing was evaluated by biomechanical testing, μCT and histomorphometrical analyses. Comparing the two groups under normoxia, the bending stiffness, moment of inertia and apparent Young’s modulus of the fractured femurs were significantly reduced by the additional thoracic trauma (-46%; p = 0.001; -33%, p = 0.032; and -43%, p = 0.021; respectively) indicating a smaller callus with poor mechanical competence (Fig 2A–2C). μCT analysis of BV/TV did not show group differences (data not shown).

**Fig 3:**
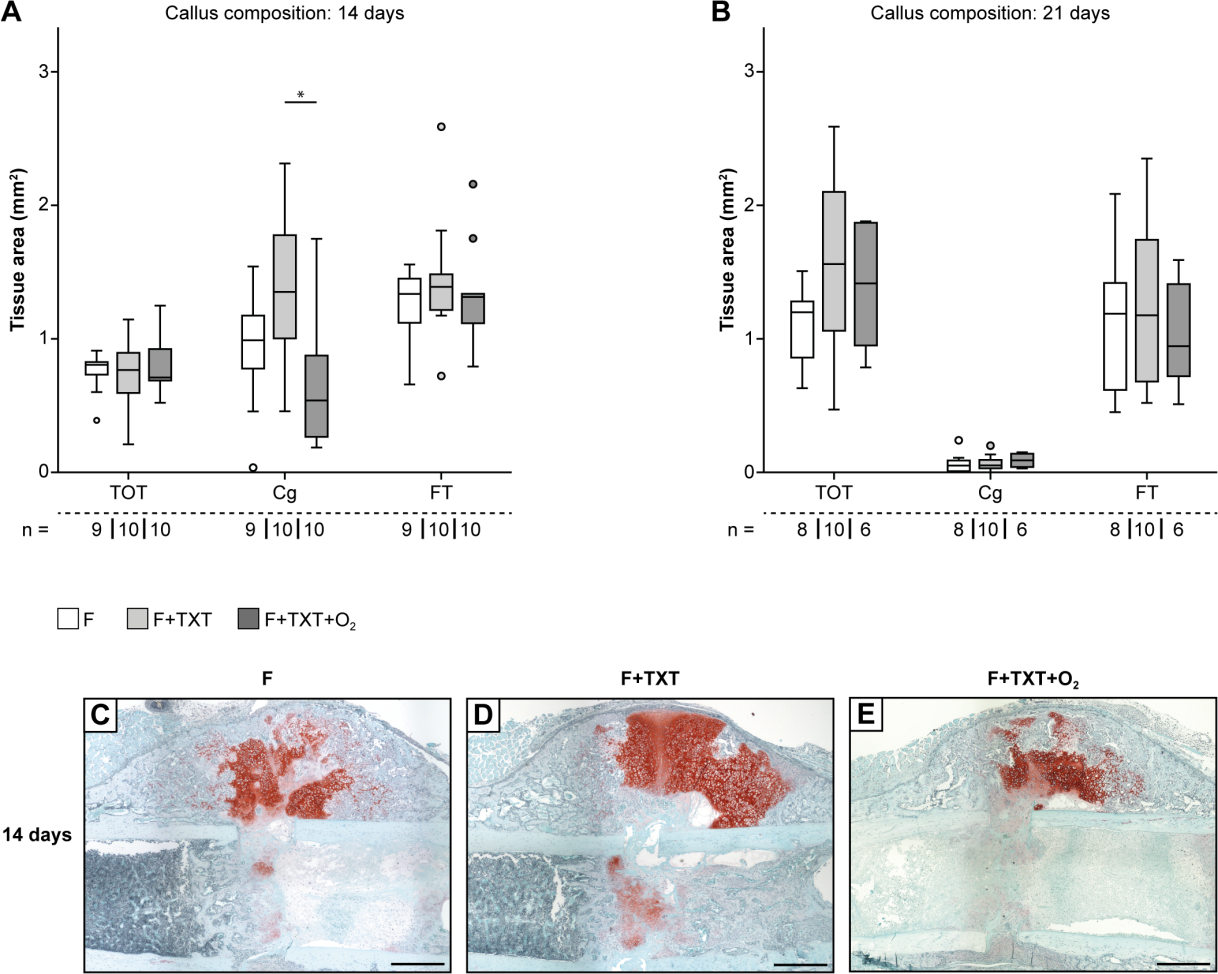
Tissue composition of fracture calli 14 and 21 days after injury. Callus composition of mice 14 and 21 days post-injury. (A) Mice with TXT displayed significantly more cartilage in comparison to O_2_ treated mice after 14 days. (B) Analysis after 21 days did not reveal intergroup differences. (C-E) Representative Safranin-O stained callus sections 14 days after injury. Markedly more cartilage (stained red) was observed in F+TXT mice compared to the other groups. TOT = total osseous tissue, Cg = cartilage, FT = fibrous tissue. Scale bars: 500 μm. Data represent medians and quartiles. Specimen numbers for each group are depicted. *p<0.05.

**Fig 4:**
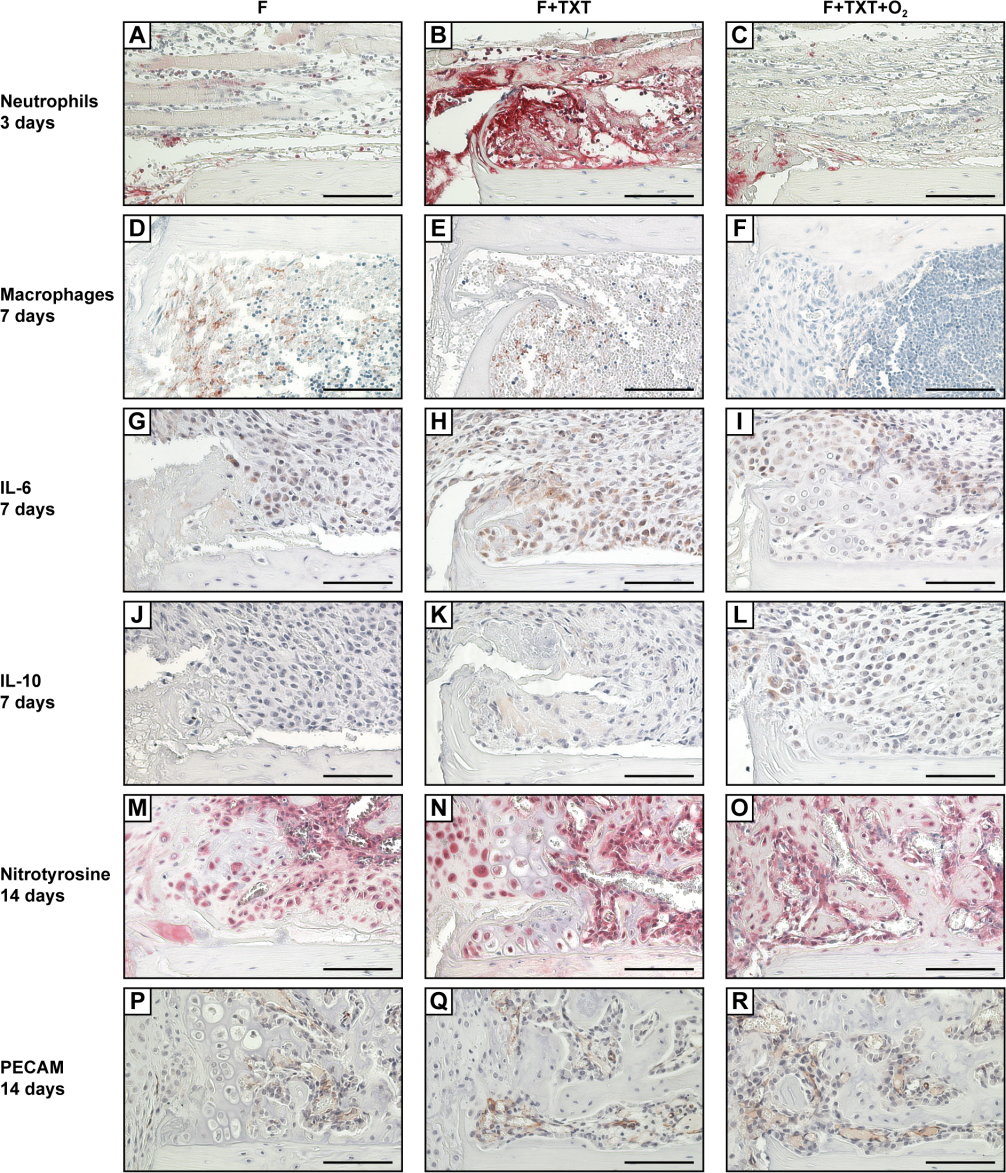
Immunohistological stainings of fractured femurs for markers of inflammation, nitrosative stress and vascularization. Left panels are representative slides of mice with isolated fracture, middle panels of animals with additional TXT, and right panels of mice with fracture, TXT and O_2_ treatment. Images indicate cortical bone proximal to the fracture gap and periosteal callus, macrophage staining show the marrow cavity. (A-C) Neutrophil staining 3 days post-injury, (D-F) macrophage staining on day 7, (G-L) IL-6 and IL-10 staining on day 7, and (M-R) nitrotyrosine and PECAM-1 staining on day 14. Scale bars: 100 μm.

Histomorphometry showed delayed endochondral ossification at 14 days in the normoxia thoracic trauma group, demonstrated in larger amounts of cartilage (Fig 3A and 3D; S1 Fig). After 21 days we did not detect histological intergroup differences in the amounts of cartilage or bone anymore (Fig 3B). Fracture calli were additionally analysed by immunohistochemistry on days 1, 3, 7 and 14 (Fig 4). In all groups, neutrophils were mainly found on day 1 and 3, while on day 7 their number had markedly decreased. After 3 days neutrophil numbers were increased in mice with thoracic trauma (Fig 4A and 4B). Thoracic trauma did not affect macrophage infiltration on day 3, high numbers were found in the marrow cavity on day 7, with no effect of chest trauma (Fig 4D and 4E). Positive IL-6 and IL-10 staining was evident on day 1, 3 and 7 without intergroup differences (Fig 4G–4L). Nitrotyrosine and PECAM-1 stainings examined on days 1, 7 and 14 did not show any intergroup differences either (Fig 4M–4R).

On the other hand, O_2_ treatment widely attenuated impaired fracture healing induced by the thoracic trauma. Bending stiffness and apparent Young’s Modulus were restored to values of the group with isolated fracture (Fig 2A and 2C). The amount of cartilage, which was increased by additional thoracic trauma, also returned to normal values (Fig 3A–3E). While thoracic trauma induced neutrophil invasion in the callus, O_2_ exposure markedly reduced neutrophil numbers on day 3 (Fig 4B and 4C). Macrophage numbers were strongly diminished after O_2_ treatment (Fig 4D and 4E). The expression of IL-6, IL-10, nitrotyrosine and PECAM-1 was not affected by O_2_ treatment.

## Discussion

The present study addressed the role of O_2_ tension in the pathophysiology of disturbed bone healing after thoracic trauma. Here, we demonstrated that a thoracic trauma induced pulmonary and systemic inflammation and impaired bone healing, even if it did not lead to sustained lung dysfunction and hypoxemia. Nevertheless, short-term exposure to 100% O_2_ in the acute post-traumatic phase significantly attenuated systemic and local inflammatory responses and, in turn, improved fracture healing, suggesting that hyperoxia could provoke anti-inflammatory and pro-regenerative effects in severe trauma and bone regeneration. These results implicate that breathing of 100% O_2_ in the acute post-traumatic phase might reduce the risk of poorly healing fractures in severely injured patients.

As expected from previous studies [11, 13, 14, 30, 31] we observed a systemic inflammatory response in mice subjected to combined thoracic trauma and femur fracture. Whereas mice with isolated fracture exhibited no changes in cytokine and chemokine levels in comparison to sham operated mice [30], the combined trauma in the normoxic group induced a significant release of pro- and anti-inflammatory mediators in the blood after 3 hours: IL-6 and IL-10 concentrations, common markers for the systemic inflammatory response in humans, which correlate with injury severity and mortality [32, 33], were significantly increased. Levels of MCP-1, a chemokine attractant for monocytes [34] were also elevated. However, arterial blood gas concentrations, acid-base status, and local haemoglobin oxygenation and microcirculatory perfusion at the fracture site were not significantly influenced indicating that the pulmonary function was not persistently affected by the thoracic trauma after 3, 9 and 24 hours. In good agreement, histo-pathological signs of lung injury, e.g. dystelectasis, alveolar wall thickening, emphysema and blood clotting, were only modest. Likewise, expression of caspase-3, a terminal apoptotic marker [35], was not significantly increased in lung tissues in the combined normoxic trauma group. However, neutrophil invasion into the lung and lung tissue levels of IL-1ß, IL-6 and IL-10 were transiently increased 3 hours after injury, indicating a pulmonary inflammatory response. Inflammation is known to activate the inducible nitric oxide (NO) synthase (NOS2) resulting in excessive NO, which in turn may react with the superoxide radical to form peroxynitrite [36]. Peroxynitrite formation can be detected due to its reaction with the ubiquitous radical amino acid tyrosine to form 3-nitrotyrosin, a stable marker, hence, of both oxidative and nitrosative stress [37]. Lung nitrotyrosine was significantly elevated after combined trauma even after 3 weeks, thus demonstrating a long-term stress response. Taken together, these data are in line with previous investigations [31] and demonstrate that our murine model of blunt chest trauma induced a transient pulmonary and systemic immune response, but did not cause sustained impairment of lung function and gas exchange. A limitation of our study might be that we did not include earlier post-traumatic observation time points. Additionally, due to a FiO_2_ of 0.35 used for the anesthesia required for the terminal blood withdrawal, we may have missed more pronounced effects, e.g. hypoxia right after trauma. Therefore, the question remains open whether pulmonary and systemic hyper-inflammation was triggered by an immediate short-term hypoxemic insult, which was already restored 3 hours after the thoracic trauma in the model used.

Confirming previous data [11, 13, 14], the present study demonstrated that an additional thoracic trauma considerably disturbed fracture healing. Mice subjected to the combined trauma displayed disturbed endochondral bone formation resulting in inferior mechanical properties in the late phase of bone healing compared to mice with isolated fracture. To investigate the question whether the thoracic trauma affected the local O_2_ supply at the fracture gap, we measured microvascular flow and haemoglobin O_2_ saturation but did not find significant alterations compared to the group with isolated fracture. This indicates that the delay of fracture healing was not primarily triggered by local O_2_ deficiency. Of note, hypoxemia did not result in vasoconstriction, because microvascular blood flow rates remained unaltered. Pure O_2_ breathing is well established to exert vasoconstrictor effects on the macro- [38] and micro-circulatory [39] level, but apparently this was not the case during hyper-inflammation.

Increased neutrophil migration to the fracture hematoma demonstrated that the post-traumatic systemic inflammation enhanced the local inflammatory response at the site of fracture confirming our previous observations in rats [13]. Neutrophils abundantly secrete reactive oxygen species and cytokines thereby acting as the first line of defence at the site of injury, while over-activated neutrophils can also cause tissue damage and delay tissue regeneration [40]. In agreement with this rational, fracture healing was improved after systemic neutrophil depletion [41]. However, nitrotyrosine was abundantly present in the fracture callus during the entire healing period no matter of the group assignment, suggesting that oxidative and nitrosative stress response was not further aggravated by the additional thoracic trauma. The number of macrophages and the local expression of cytokines, such as IL-6 and IL-10, were not significantly affected by the thoracic trauma. This finding is in contrast to the previous rat study [13], possibly due to species differences. Nevertheless, our data demonstrate that the systemic post-traumatic inflammation induced by the thoracic trauma disturbs the inflammatory response in the fracture hematoma and thus impairs fracture healing without persistently affecting local O_2_ supply.

Albeit the thoracic trauma did not induce sustained lung dysfunction and hypoxemia in our murine model, we were interested if short-term O_2_ therapy could ameliorate pulmonary and systemic inflammation and thus improve fracture healing. It has been shown before, that transient hyperoxia could exert anti-inflammatory and anti-apoptotic effects, e.g. in ischemia/reperfusion injury [21, 42], cecal-ligation-and-puncture induced peritonitis [43, 44], or septic shock [20, 45], which resulted in improved organ function and, ultimately, survival. Due to its beneficial effects, O_2_ is even regarded as an “anti-inflammatory drug” [46–48]. On the other hand, the toxic effects of hyperoxia are well established. Hyperoxia can aggravate oxidative and nitrosative stress and thereby cause hyper-inflammation [22, 49]. However, harmful effects were only observed after long-term exposition to 100% O_2_ > 24 hours [50] or in combination with injurious mechanical lung ventilation [51, 52]. In contrast, toxic effects were not observed after short-term exposition to 100% O_2_ in the acute phase of severe trauma or shock [46–48]. Based on the beneficial findings of previously published studies in sepsis models [43, 44], we selected a short-term intermittent exposure to 100% O_2_.

Hyperoxia did not provoke any apparent toxic side effects, as indicated by low levels of DNA-strand breaks and the unchanged expression of HO-1, which is induced by oxidative and nitrosative stress as part of the cellular defence system to ameliorate oxidative damage and apoptosis [53, 54]. Moreover, hyperoxia attenuated lung and systemic inflammation as confirmed by the significantly reduced neutrophil invasion into the lung and diminished pulmonary and systemic levels of pro- and anti-inflammatory cytokines. Furthermore, the otherwise increased lung nitrotyrosine formation was attenuated, indicating that inhalation of 100% O_2_ counteracted oxidative and nitrosative stress induced by the thoracic trauma under normoxia.

Notably, hyperoxia nearly completely abolished the deleterious effects of the combined trauma on fracture healing. The cartilage fraction in the callus, which was increased in the combined trauma group under normoxic conditions, was significantly diminished after O_2_ treatment indicating reduced cartilage formation and/or accelerated cartilage-bone transformation. In the later healing phase the mechanical properties of the fracture callus were increased indicating a more mature callus with improved tissue quality. *In vitro* data suggest that O_2_ tension regulates stem cell differentiation. It was shown that the differentiation of mesenchymal precursor cells toward the chondrogenic lineage was favoured in a hypoxic environment [55, 56]; in contrast, osteogenic differentiation was improved by increasing the O_2_ tension [57, 58]. In our study, short-term exposure to 100% O_2_ significantly enhanced capillary haemoglobin O_2_ saturation locally at the fracture site, but only after 3 hours. Because the fracture hematoma is primarily dominated by immune cells at this early time point [59], we suggest that the improvement of bone regeneration may probably rather be caused by the modulation of local inflammatory processes than by promoting osteogenesis of precursor cells, which invade the fracture hematoma at a later time point. In good agreement with this hypothesis, O_2_ treatment significantly decreased the numbers of neutrophils and macrophages in the fracture hematoma. Because long-term O_2_ exposition was shown to increase vascularization in fracture healing [60] we performed immunostaining for PECAM-1, a marker for angiogenesis [61]. Blood vessel formation in the fracture callus was not affected by short-term O_2_ treatment indicating that improved bone formation was not associated with higher vascularity. Taken together, transient exposure to 100% O_2_ in the acute phase after thoracic trauma attenuated the pulmonary and systemic inflammatory response and, in turn, local inflammatory processes at the site of fracture thus improving bone regeneration.

To our knowledge this is the first study to investigate the effect of short-term 100% O_2_ application on fracture healing in severe trauma. So far, some pre-clinical and clinical studies investigated the effect of hyperbaric [62–67] or normobaric O_2_ therapy [60] on bone healing reporting positive [63–67], no [62], or controversial effects in different models [60]. Hyperbaric O_2_ therapy, e.g. intermittently administering 100% O_2_ at supra-atmospheric pressures, may bear the risk of side effects, including pulmonary- and central neurotoxicity [47], and is critically discussed for the treatment of poorly healing fractures [62, 68]. To our knowledge, so far only one study investigated the effect of normobaric O_2_ application on bone healing [60]. The authors applied 50% O_2_ during the entire period in a mouse model of uncomplicated fracture healing and in ischemia-induced delayed fracture union. Hyperoxia improved bone repair only under compromised conditions [60]. Even if long-term O_2_ application may be obsolete due to toxic side effects, these results, in accordance to ours, suggest a positive effect of O_2_ treatment under compromised healing conditions. However, a limitation of the present study might be that we did not investigate the effect of O_2_ treatment in the isolated fracture model.

In conclusion, our results implicate that short-term breathing of 100% O_2_ in the acute post-traumatic phase might reduce the risk of poorly healing fractures in patients with additional thoracic trauma. Further studies are necessary to clarify if O_2_ treatment is also useful in other patterns of severe injures, to fully understand the underlying molecular mechanisms of O_2_ on bone regeneration, and also to prove the therapeutic effect of O_2_ short-term treatment in humans.

## Supporting Information

S1 Fig Tissue composition of fracture calli 14 days after injury. The figure shows histological images, stained with Safranin-O of all mice 14 days post-injury for the corresponding treatment groups: F = isolated fracture; F + TXT = fracture + thoracic trauma; F + TXT + O_2_ = fracture + thoracic trauma + O_2_ treatment. Scale bars: 500 μm.
(TIF)
