# Supplementary figures and images for: Exposure to 100% Oxygen Abolishes the Impairment of Fracture Healing after Thoracic Trauma
Source: PLoS One. 2015 Jul 6;10(7):e0131194. doi: 10.1371/journal.pone.0131194 (PMC4492600; doi:10.1371/journal.pone.0131194)

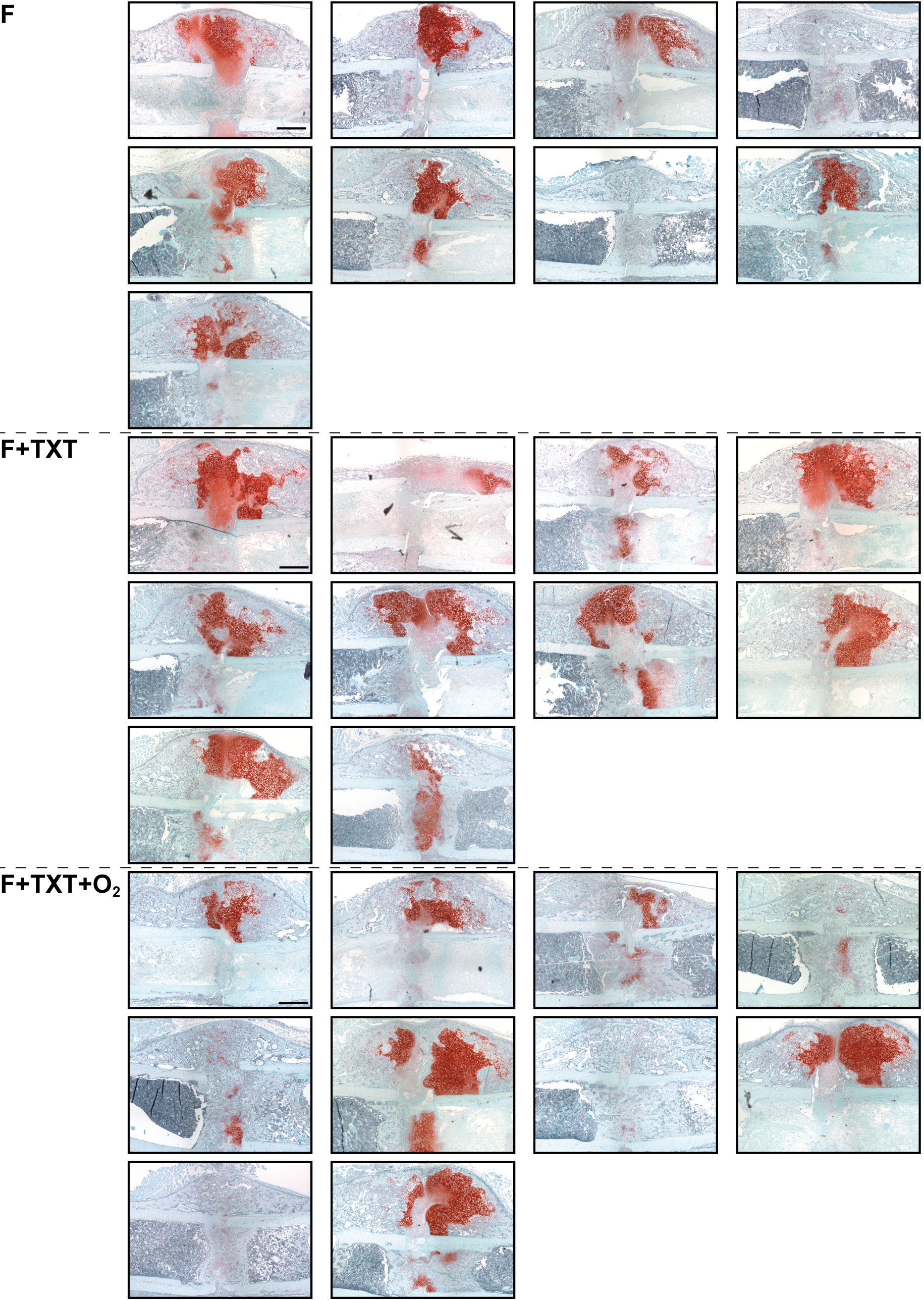

Supplement: S1 Fig — The figure shows histological images, stained with Safranin-O of all mice 14 days post-injury for the corresponding treatment groups: F = isolated fracture; F + TXT = fracture + thoracic trauma; F + TXT + O2 = fracture + thoracic trauma + O2 treatment. Scale bars: 500 μm. (TIF) [file pone.0131194.s001.tif]
